# Antimicrobial and anticancer activity of some novel fluorinated thiourea derivatives carrying sulfonamide moieties: synthesis, biological evaluation and molecular docking

**DOI:** 10.1186/s13065-017-0258-4

**Published:** 2017-04-07

**Authors:** Mostafa M. Ghorab, Mansour S. Alsaid, Mohamed S. A. El-Gaby, Mahmoud M. Elaasser, Yassin M. Nissan

**Affiliations:** 1 Pharmacognosy Department, College of Pharmacy, King Saud University, P.O. Box 2457, Riyadh, 11451 Saudi Arabia; 2 Department of Drug Radiation Research, National Center for Radiation Research and Technology, Nasr City, Cairo, 113701 Egypt; 3 Department of Chemistry, Faculty of Science, Al-Azhar University at Assiut, Assiut, 71524 Egypt; 4 The Regional Center for Mycology and Biotechnology, Al-Azhar University, Cairo, Egypt; 5 Department of Pharmaceutical Chemistry, Faculty of Pharmacy, Cairo University, Cairo, Egypt

**Keywords:** Isothiocyanate, Sulfonamide, Fluorinated thiourea, Antimicrobial and anticancer activity

## Abstract

**Background:**

Various thiourea derivatives have been used as starting materials for compounds with better biological activities. Molecular modeling tools are used to explore their mechanism of action.

**Results:**

A new series of thioureas were synthesized. Fluorinated pyridine derivative **4a** showed the highest antimicrobial activity (with MIC values ranged from 1.95 to 15.63 µg/mL). Interestingly, thiadiazole derivative **4c** and coumarin derivative **4d** exhibited selective antibacterial activities against Gram positive bacteria. Fluorinated pyridine derivative **4a** was the most active against HepG2 with IC50 value of 4.8 μg/mL. Molecular docking was performed on the active site of MK-2 with good results.

**Conclusion:**

Novel compounds were obtained with good anticancer and antibacterial activity especially fluorinated pyridine derivative **4a** and molecular docking study suggest good activity as mitogen activated protein kinase-2 inhibitor.

**Graphical abstract:**
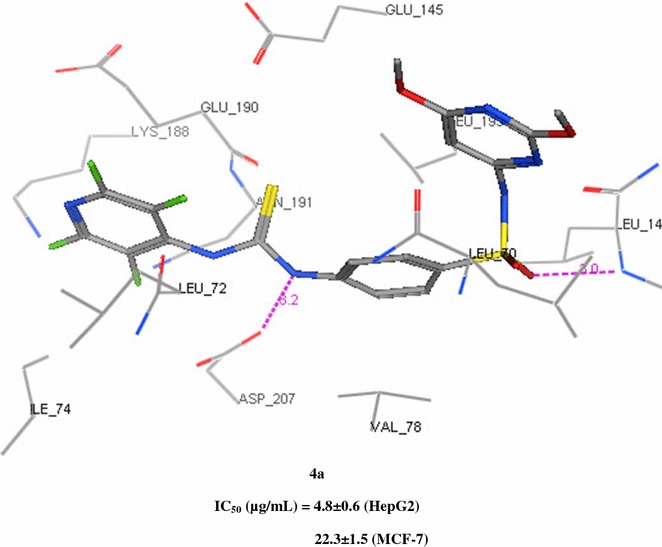
Compound 4a in the active site of MK-2

## Background

**Fig. 1:**
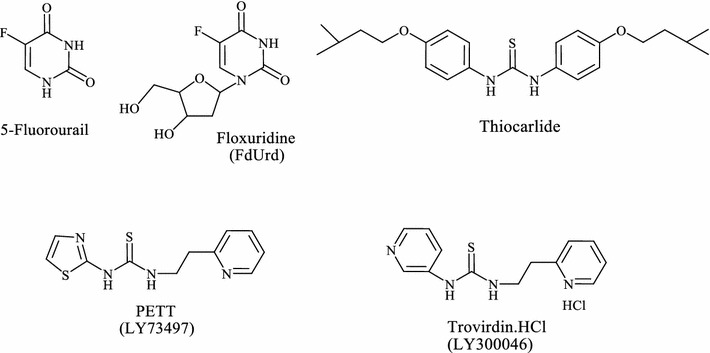
Fluorinated and thiourea anticancer agents

Fluorinated compounds are intriguing for the development of pharmaceuticals, agrochemicals, and materials, and thus, much effort has been exerted to develop more general and efficient approaches for introducing fluorine atom(s) or fluoroalkyl group(s) into organic molecules [1–4]. The unique properties of fluoro organic molecules may arise from the properties such as (i) the greatest electronegativity of fluorine, (ii) the largest strength of the carbon–fluorine bond, (iii) the hardness and the low van der Waals interaction due to the low polarizability, (iv) the increased hydrophobicity, and (v) the second smallest atomic radius of the fluorine atom. These factors are operative singly or sometimes cooperatively to affect the pharmacological properties of the fluorinated molecules [5]. The majority of fluorinated drugs are constructed by five- and six-membered nitrogen heterocycles containing fluorine, trifluoromethyl, difluoromethyl, fluoromethyl, 2,2,2-trifluoroethyl, and pentafluoroethyl groups [6]. An increasing number of fluorinated antimitotic/antitumour agents have now becoming available for cancer treatment. The most widely used are the 5-fluoropyrimidines such as 5-fluorouracil (5-FU) and 5-fluoro-2^\^-deoxyuridine (FdUrd) [7, 8], (Fig. 1). The thiourea derivatives represent one of the most promising classes of anticancer agents with a wide range of activities against various leukemia and solid tumors [9–17]. They play an important role as anticancer agents because of their good inhibitory activity against protein tyrosine kinases (PTKs), [10–13] human sirtuin type proteins 1 and 2 (SIRT1 and SIRT2), [14] topoisomerase II [15] and DNA repair synthesis [16]. Furthermore, fluorinated aryl thioureas represent a new class of potent anti-trypanosomal agents [18] and also a novel class of potent influenza virus neuraminidase inhibitors [19]. Thiocarlide is a pharmacologically important thiourea drug that is used as a therapeutic agent in the treatment of tuberculosis [20] and Phenethylthiazoylthiourea (PETT) derivatives (LY73497 and trovirdine HCl) [21, 22] have been discovered as potent inhibitors of HIV type 1, (Fig. 1).

Literature survey revealed that sulfonamides are a significant class of compounds in medicinal and pharmaceutical chemistry with several biological applications [23]. Today, they are widely used as antimicrobial agent, chiefly because of their low cost, low toxicity and excellent activity against bacterial diseases [24]. Some important sulfonamide derivatives used as carbonic anhydrase inhibitors of commercial importance [25]. They are also effective for the treatment of urinary, intestine, and ophthalmic infections, scalds, ulcerative colitis [26], rheumatoid arthritis [27], male erectile dysfunction as the phosphodiesterase-5 inhibitor sildenafil-better known under its commercial name, Viagra [28], and obesity [29]. More recently, sulfonamides are used as an anticancer agent [30], as the antiviral HIV protease inhibitor amprenavir [31] and in Alzheimer’s disease [32]. Prompted by the above facts and in continuation of our interest in biologically active compounds [33–35] we hereby report the synthesis of some novel of fluorinated *N*-(2,6-dimethoxypyrimidin-4-yl)-4-(3-(aryl)thioureido)benzenesulfonamides **3a**–**e** and **4a**–**d** from readily available starting material to evaluate their antimicrobial and anticancer activity.

## Results and discussion

### Chemistry

**Scheme 1:**
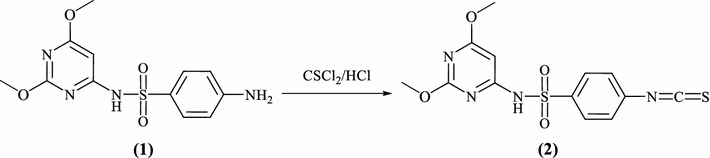
Synthesis of *N*-(2,6-dimethoxypyrimidin-4-yl)-4-isothiocyanato-benzenesulfonamide **2**

Isothiocyanates are useful and widely used building blocks in the synthesis of nitrogen, sulfur and oxygen heterocycles and organometallic compounds of academic, pharmaceutical and industrial interest [36]. The high electrophilicity and nucleophilicity associated with the carbon and sulfur atoms, respectively, of the isothiocyanates and their extended π electron system make them unique precursors of a large variety of target molecules. Consequently, many classes of five and six-membered nitrogen and sulfur heterocycles, either carrying various substituents or fused with benzo or non-benzo nuclei to interesting poly heterocycles, have been synthesized from isothiocyanates which is undoubtedly a landmark in organosulfur chemistry [37]. The intermediate, *N*-(2,6-dimethoxypyrimidin-4-yl)-4-isothiocyanatobenzenesulfonamide **2** used for the preparation of target compounds have been synthesized in high yield via thiophosgenation of sulfa-dimethoxazine **1** at room temperature in the presence of dilute hydrochloric acid, according to literature procedure [38] (Scheme 1).

**Scheme 2:**
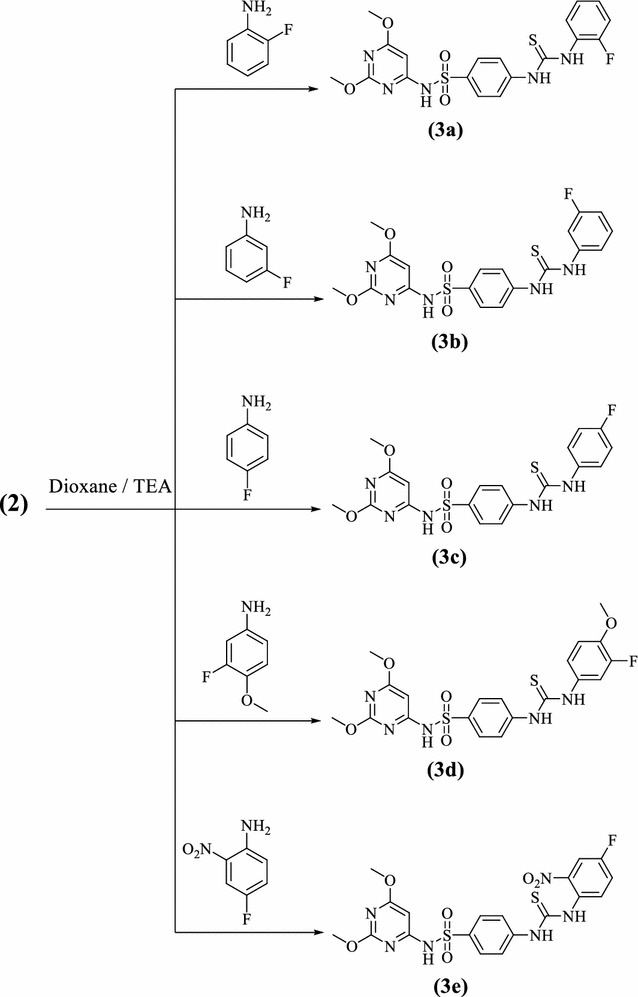
Synthetic route and structures for thiourea derivatives (**3a**–**e**)

**Fig. 2:**
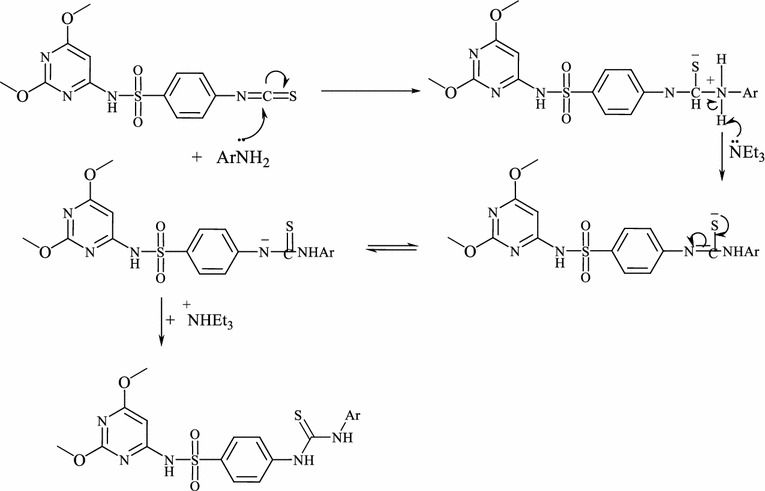
The proposed mechanism for the formation of thiourea

The synthesis of *N*-(2,6-dimethoxypyrimidin-4-yl)-4-(3-(aryl) thio-ureido)benzenesulfonamides **3a**–**e** is outlined in Scheme 2. Treatment of isothiocyanato benzenesulfonamide **2** with a variety of fluorinated aromatic amines in dry dioxane at reflux temperature in the presence of a catalytic amounts of triethylamine furnished the novel fluorinated *N,N*-disubstituted thioureas **3a**–**e** in high yields (80–92%).The structure of the products **3a**–**e** were established via inspection of their spectral data. Thioureas **3a**–**e** were confirmed by the absence of characteristic infrared absorption peak at 2000–2200 cm^−1^ (N=C=S group). Also, the infrared of **3** is characterized by the presence of the NH, CN, thiocarbonyl (CS) and SO_2_ absorption bands. For example, the ^1^H NMR of compound **3a** showed two singlets at *δ* 3.81, 3.84 ppm which were assigned for two methoxy protons, a singlet at *δ* 5.9 ppm assigned to the pyrimidine-H, two downfield singlets at *δ* 11.8, and 14.0 ppm which were readily assigned to the HN(1) and HN(2) protons, in addition to the presence of SO_2_NH and aromatic protons. The thiocarbonyl group of thiourea moiety was also observed in ^13^C-NMR. The formation of thiourea **3a**–**e** can be explained by the reaction pathway depicted in Fig. 2.

**Scheme 3:**
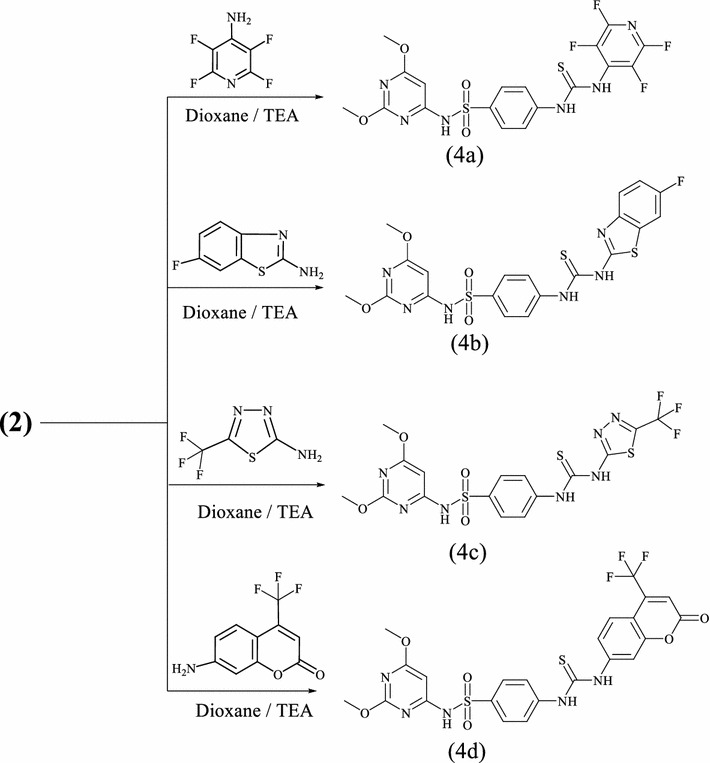
Synthetic route and structures for thiourea derivatives (**4a**–**d**)

The nucleophilic attack of the amino group of the aromatic amine on thiocarbonyl group of isothiocyanate leads to formation of an intermediate (**A**). During the consecutive steps, deprotonation and protonation of the intermediate results in the formation of the final product thiourea. Under similar reaction conditions, treatment of isothiocyanate **2** with fluorinated heterocyclic amines such as 2-amino-2,3,5,6-tetrafluoropyridine, 2-amino-6-fluorobenzothiazole, 2-amino-5-(trifluoromethyl)-1,3,4-thiadiazole and 7-amino-4-(trifluoromethyl)-coumarine afforded the corresponding fluorinated heterocyclic thioureas **4a**–**d**, (Scheme 3). The composition and structure of products **4a**–**d** were confirmed by the results of elemental analysis and data of IR and NMR spectra. The infrared of structure **4** displayed absorption band assignable for NH, thiocarbonyl (CS) and SO_2_ groups. The infrared of **4c** exhibited stretching frequencies at 3415, 3310, 2978, 2841 and 1618 cm^−1^ for the two NH, CH-aliph and CN groups, in addition to the presence of absorption bands corresponding to SO_2_ and CS at 1311, 1195, 1274 cm^−1^. Its ^1^H NMR showed two singlets at *δ* 3.64, 3.66 ppm which were assigned for two methoxy protons, a singlet at *δ* 6.5 ppm assigned to the pyrimidine-H, two downfield singlets at *δ* 11.8, and 12.4 ppm which were readily assigned to the HN(1) and HN(2) protons, in addition to the presence of SO_2_NH and aromatic protons (Scheme 3).

### Antimicrobial evaluation

The newly synthesized target compounds were evaluated for their in vitro antibacterial activity against *Streptococcus pneumoniae* and *Bacillus subtilis* as examples of Gram-positive bacteria and *Pseudomonas aeruginosa* and *Escherichia coli* as examples of Gram-negative bacteria. They were also evaluated for their in vitro antifungal potential against a representative panel of fungal strains i.e. *Aspergillus fumigatus*, and *Candida albicans*. The organisms were tested against the activity of solutions of concentrations (1 mg/mL) and using inhibition zone diameter in mm as criterion for the antimicrobial activity (agar well diffusion method). The results of testing for antibacterial and antifungal effects are summarized in Table 1. As shown by these results, the newly synthesized compounds tested displayed variable in vitro antibacterial and antifungal activities.

**Table 1:** In vitro antimicrobial activities of the synthesized fluorinated compounds tested at 1 mg/mL by well diffusion agar assay and expressed as inhibition zone diameter (mm) in the form of mean ± SD

Tested microorganisms compound code	Fungi		Gram positive bacteria		Gram negative bacteria	
	*A. fumigatus* RCMB 002568	*C. albicans* RCMB 005036	*S. pneumoniae* RCMB 010010	*B. subtilis* RCMB 010067	*P. aeruginosa* RCMB 010043	*E. coli* RCMB 010052
**3a**	0	0	0	0	0	0
**3b**	0	0	0	0	0	0
**3c**	0	0	0	0	0	0
**3d**	16.9 ± 1.2	0	15.4 ± 0.8	18.2 ± 0.6	12.4 ± 0.8	15.1 ± 1.2
**3e**	0	0	0	0	0	0
**4a**	20.1 ± 1.3	0	20.6 ± 1.5	22.1 ± 1.2	17.2 ± 1.5	21.3 ± 0.8
**4b**	16.3 ± 0.5	0	14.2 ± 0.4	17.1 ± 0.6	11.2 ± 0.7	13.4 ± 0.7
**4c**	0	0	15.1 ± 0.7	19.8 ± 1.1	0	0
**4d**	0	0	16.3 ± 1.2	18.3 ± 0. 8	0	0
Amphotricin B	23.7 ± 1.2	25.4 ± 1.1				
Ampicillin			23.8 ± 0.6	26.4 ± 0.7		
Gentamycin					19.7 ± 0.6	24.9 ± 1.5

From the screening results, it can be seen that compound **4a** showed the highest activity against Gram positive bacteria *B. subtilis* followed by compounds **4c**, **4d**, **3d** and **4b**, respectively. Similarly, compound **4a** showed the highest activity against Gram positive bacteria *S. pneumoniae* followed by compounds **4d**, **3d**, **4c**, and **4b**, respectively using ampicillin as reference drug. Compound **4a** showed inhibition zone of 20.6 ± 1.5 mm in case of *S. pneumoniae* compared to inhibition zone of 23.8 ± 0.6 mm attributed to ampicillin while in case of *B. subtilis,* compound **4a** showed inhibition zone of 22.1 ± 1.2 mm compared to inhibition zone of 26.4 ± 0.7 mm due to ampicillin. On the other hand, compound **4a** showed the highest activity against Gram negative bacteria (*P. aeruginosa* and *E. coli*) compared with the standard drug followed by compounds **3d**, and **4b**, respectively. Compound **4a** showed inhibition zone of 17.2 ± 1.5 mm in case of *P. aeruginosa* compared to inhibition zone of 19.7 ± 0.6 mm attributed to gentamycin while in case of *E. coli,* compound **4a** showed inhibition zone of 21.3 ± 0.8 mm compared to inhibition zone of 24.9 ± 1.5 mm due to gentamycin. Interestingly, compounds **4c** and **4d** exhibited selective antibacterial activities against Gram positive bacteria.

Regarding the activity of the tested compounds against the tested filamentous fungus *A. fumigatus*, the order of activity **4a**, **3d**, and **4b**. No antimicrobial activities were detected for compounds **3a**, **3c** and **3e**. Also, none of the tested compound exerts any activity against the pathogenic yeast species **(** *C. albicans*) under these screening conditions. Compound **4a** was the most active compound in this case also. Compound **4a** showed inhibition zone of 20.1 ± 1.3 mm compared to 23.7 ± 1.2 mm exhibited by amphotrecin B. The antimicrobial activities of the most active synthesized fluorinated compounds were also tested to determine the minimum inhibitory concentration (Table 2). Moreover, compound **4a** showed the highest activity (MIC values ranged from 1.95 to 15.63 µg/mL), followed by **3d** (MIC 7.81–250 µg/mL) and **4b** (MIC 7.81–250 µg/mL).

**Table 2:** The antimicrobial activities of the most active synthesized fluorinated compounds expressed as minimum inhibitory concentration (µg/mL)

Compound code	*A. fumigatus* RCMB 002568	*S. pneumoniae* RCMB 010010	*B. subtilis* RCMB 010067	*P. aeruginosa* RCMB 010043	*E. coli* RCMB 010052
**3d**	15.63	62.5	7.81	250	62.5
**4a**	3.9	3.9	1.95	15.63	3.9
**4b**	62.5	125	15.63	250	125
Amphotricin B	1.95	–	–	–	–
Ampicillin	–	0.98	0.49	–	–
Gentamycin	–	–	–	3.9	0.98

### Structure activity relationship

Regarding activity against Gram positive bacteria, mono substituted fluorophenyl derivatives **3a**–**3c** showed no activity also the nitro fuolorinated derivative **3e** was also inactive. The best activity was attributed to tetrafluoro pyridine derivative **4a** indicating that increasing the number of fluoro substitutions has good impact on activity followed by trifluoromethyl derivatives **4c** and **4d** with tri fluoro substitution and finally the fluoro methoxy derivative **3d** and the fluorinated benzothiazole derivative **4b**. Similarly, The tetrafluoro pyridine derivative **4a** was the most active compound on Gram negative bacteria. Also in case of antifungal activity the tetrafluoro pyridine derivative **4a** was the most active compound.

### Cytotoxic activity

**Fig. 3:**
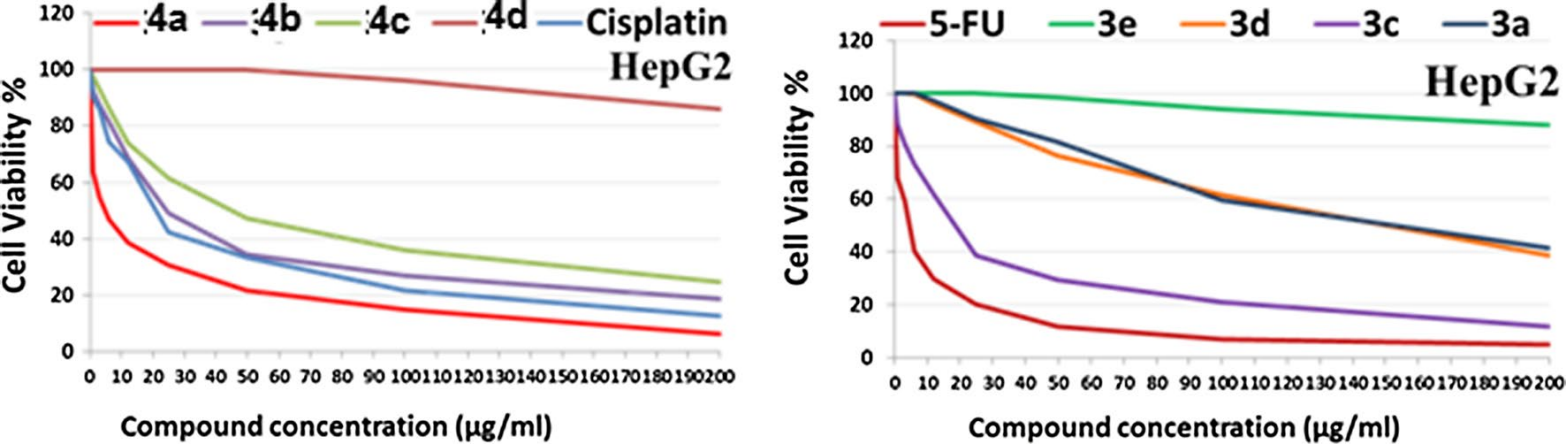
The dose response curve showing the in vitro inhibitory activity of the tested compounds against liver carcinoma (HepG2) cell line compared with reference drugs cisplatin and 5-flourouracil

The in vitro growth inhibitory activity of the synthesized compounds was investigated in comparison with the well-known anticancer standard drugs (5-flourouracil and cisplatin) under the same conditions using colorimetric MTT assay. Data generated were used to plot a dose response curve of which the concentration of test compounds required to kill 50% of cell population (IC_50_) was determined (Fig. 3). The results revealed that all the tested compounds showed inhibitory activity to the tumor cell lines in a concentration dependent manner. Cytotoxic activity was expressed as the mean IC_50_ of three independent experiments.

**Fig. 4:**
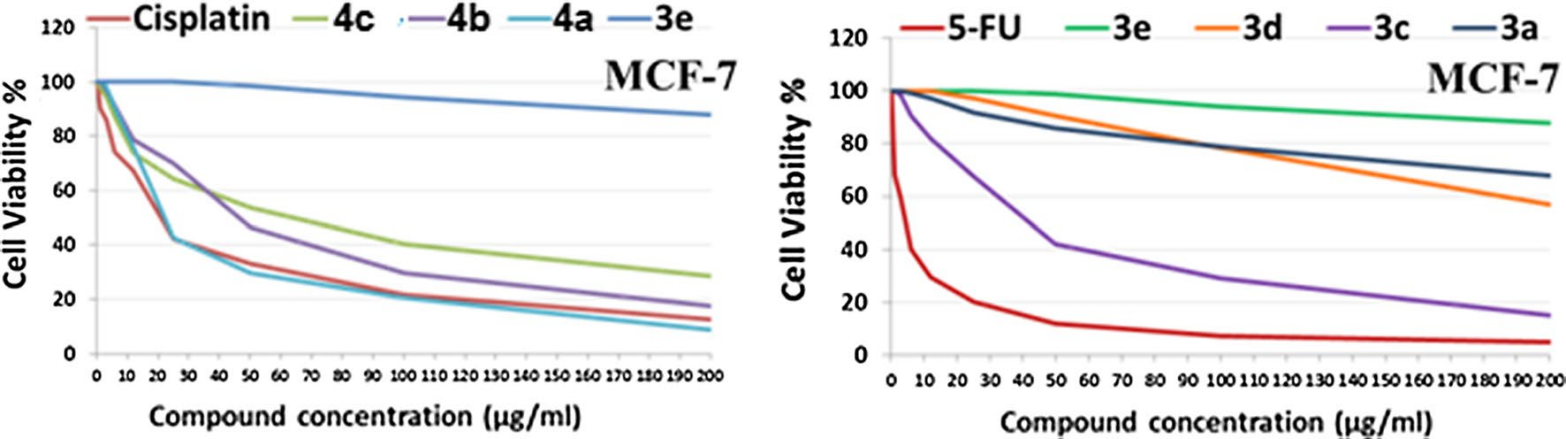
The dose response curve showing the in vitro inhibitory activity of the tested compounds against breast carcinoma (MCF-7) cell line compared with reference drugs cisplatin and 5-flourouracil

Interestingly, the results are represented in Table 3 and Fig. 3 showed that compound **4a** was the most active against the liver carcinoma cell line (HepG2), showing more activity than the reference drugs with IC_50_ value of 4.8 μg/mL compared to 5-flourouracil with IC_50_ value of 4.9 μg/mL and cisplatin with IC_50_ value of 18.8 μg/mL. Compound **3c** exhibited good antitumor activity against the liver carcinoma cell line (HepG2) showing almost the same activity as cisplatin followed by **4b**, **4c**, **3d** and **3a**, respectively. The tested compounds showed lower tendency to inhibit the breast carcinoma cells than those observed for liver carcinoma (Fig. 4). The order of activity against breast carcinoma cell line (MCF-7) was **4a**, **3c**, **4b**, and **4c**, respectively. Moreover, compounds **3a**, **3d**, **3e** and **4d** were less active among their analogues against the two tumor cell lines.

**Table 3:** The antitumor activities of the tested compounds expressed as IC_50_ values and compared with reference standard drugs evaluated on breast and liver cancer cell lines

Tested compounds	IC_50_ values (µg/mL) against tumor cell lines	
	MCF-7	HepG2
**3a**	>200	153.1 ± 2.3
**3b**	NA	NA
**3c**	41.9 ± 1.7	18.8 ± 1.1
**3d**	>200	150.9 ± 3.7
**3e**	>200	>200
**4a**	22.3 ± 1.5	4.8 ± 0.6
**4b**	46.1 ± 1.6	24.5 ± 1.2
**4c**	63.8 ± 1.2	45.2 ± 1.4
**4d**	>200	>200
5-flourouracil	5.2 ± 0.5	4.9 ± 0.3
Cisplatin	19.1 ± 0.7	18.8 ± 0.6

### Molecular docking

**Fig. 5:**
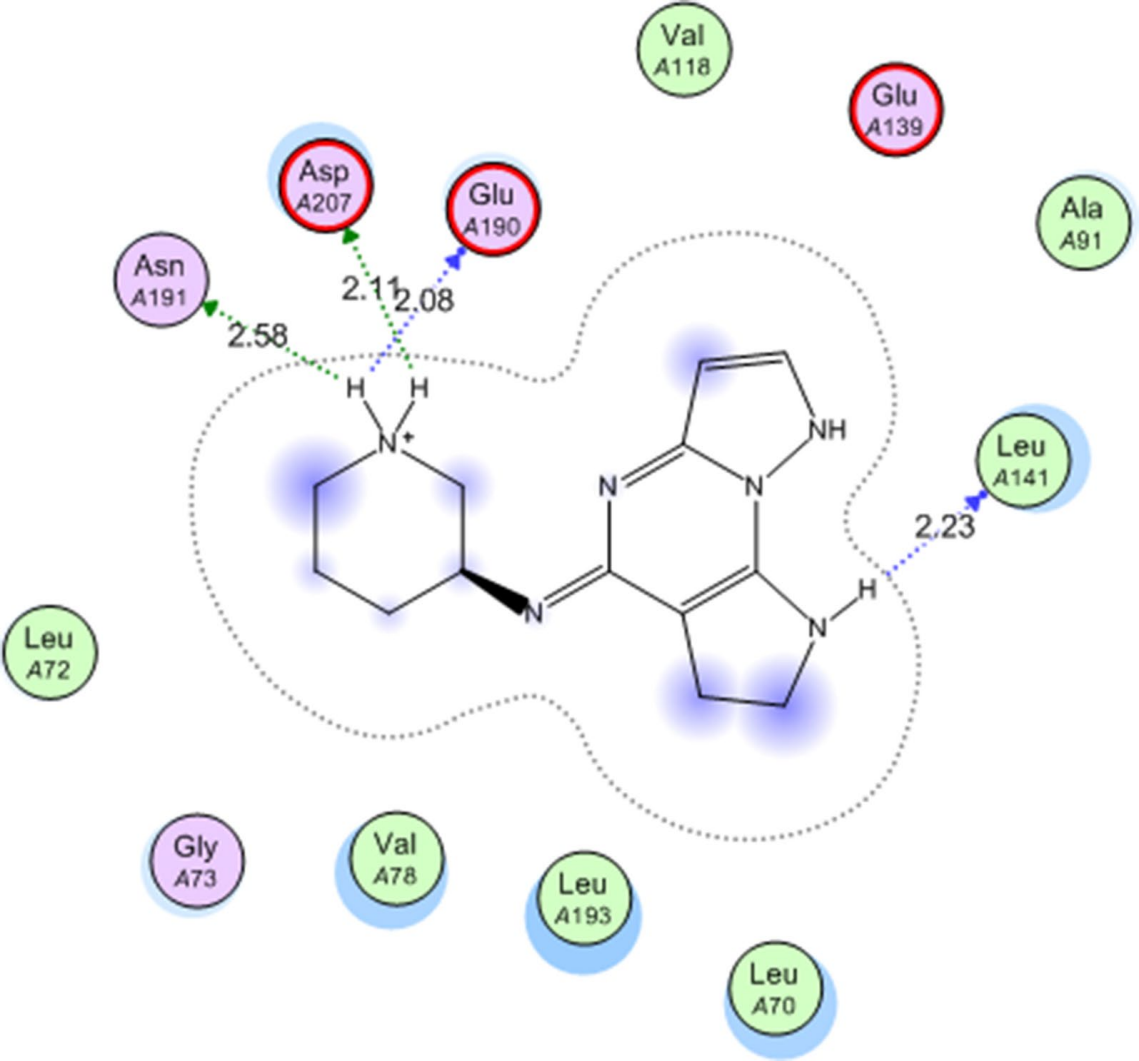
Co-crystallized ligand in the active site of mitogen activated kinase (MK-2)

One of the most important enzymes that controls signal transduction and cell proliferation is mitogen-activated protein kinase-activated protein kinase 2 (MAPKAPK-2 or MK-2) [39]. Discovering new inhibitors for this key enzyme has received attention as a strategy in the seek for novel anticancer agents [40]. Among the newly discovered inhibitors for this enzyme, several urea and thiourea derivatives have showed good activity [41]. In the present research, several thiourea derivatives were synthesized and evaluated for their cytotoxic activity. The most active derivatives **3c** and **4a**–**4c** were docked on the active site of MK-2 enzyme in a trial to suggest a mechanism of action for their cytotoxic activity. The protein data bank file (PDB: 3WI6). The file contains MK-2 enzyme co-crystalized with an inhibitor. All docking procedures were achieved by MOE (Molecular Operating Environment) software 10.2008 provided by chemical computing group, Canada. The inhibitor interacts with MK-2 active site with four hydrogen bonds involving Glu 190, Leu 141, Asn 191 ans Asp 207 (Fig. 5). The docking protocol was validated by redocking of the ligand on the active site of MK-2 enzyme with energy score (S) = −15.4978 kcal/mol and root mean square deviation (RMSD) = 1.1457.

The previous docking protocol was followed for compounds **3c** and **4a**–**4c**. All the docked compounds were fit on the active site of MK-2 enzyme. Docking scores and amino acid interactions for the docked compounds were summarized in Table 4.

**Table 4:** Docking scores and amino acid interactions of the docked compounds on the active site of mitogen activated kinase (MK-2)

Compounds	(S) Kcal/mol	Amino acid	Interacting group	Type of interaction	H-bond length (Å)
**3c**	−18.8805	Asp 207	NH	H bond (donor)	1.25
**4a**	−20.8063	Leu 141 Asp 207	SO_2_ NH	H bond (acceptor) H bond (donor)	2.96 2.22
**4b**	−16.2293	Asp 207	NH	H bond (donor)	2.12
**4c**	−22.9000	Asp 207	NH	H bond (donor)	1.53

**Fig. 6:**
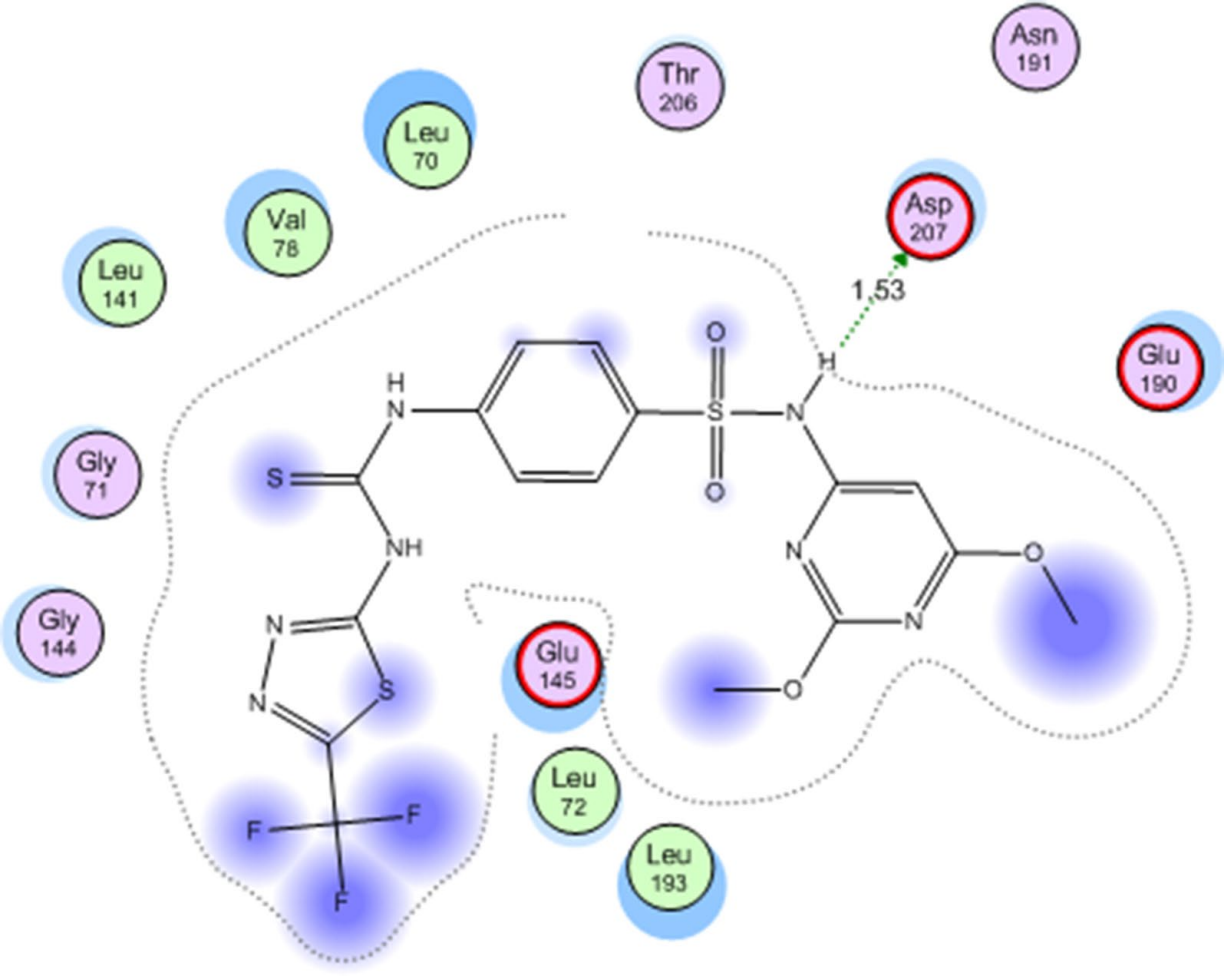
Compound **4c** in the active site of mitogen activated kinase (MK-2)

On a closer look on Table 4 we can conclude that: all four compounds showed docking score better than the co-crystallized ligand in the range of (−16.2293 to 22.9000 kcal/mol). The best docking score was displayed by compound **4c** (Fig. 6).

**Fig. 7:**
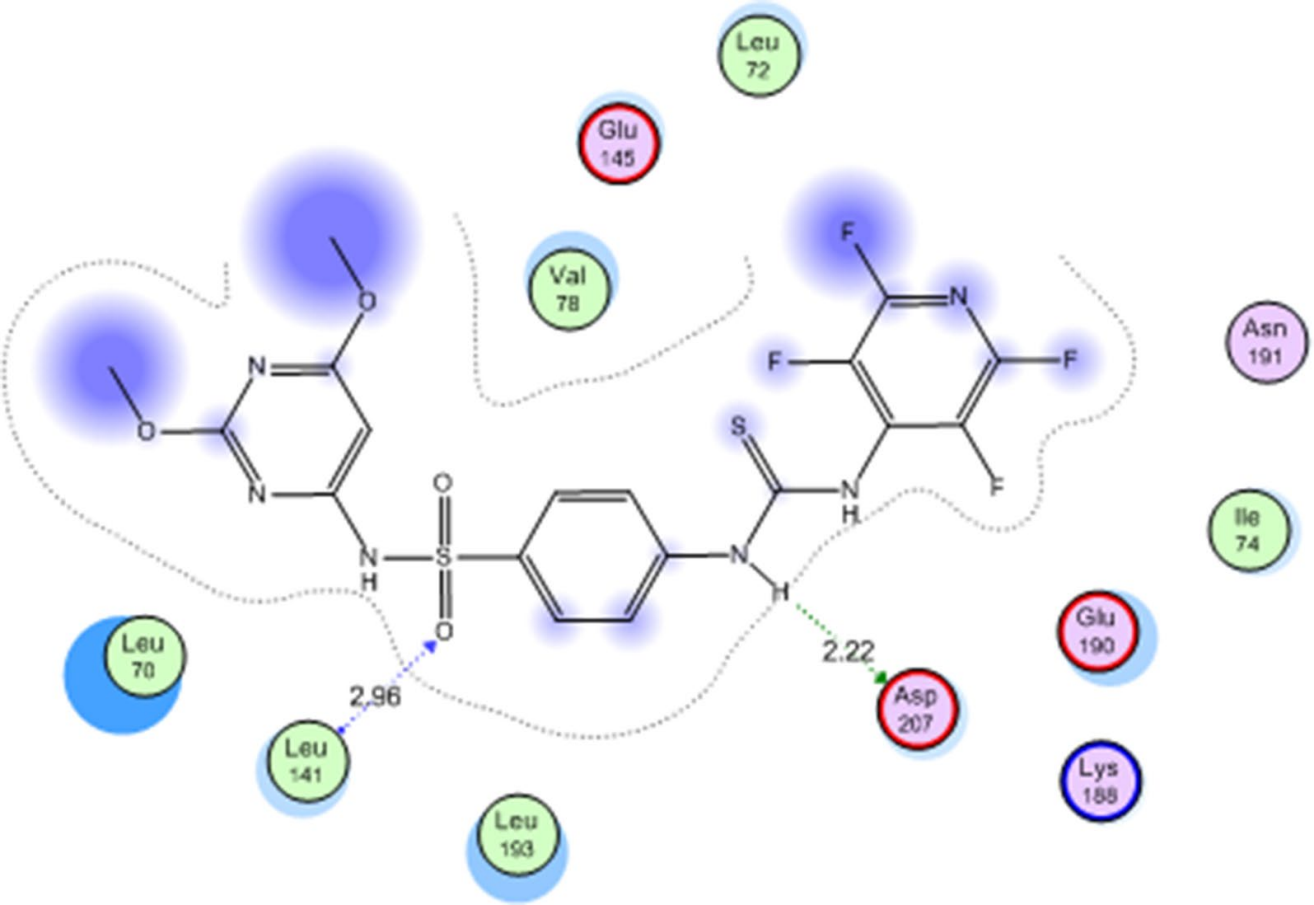
Compound **4c** in the active site of mitogen activated kinase (MK-2)

In case of amino acid interactions, compound **4a** is the only compound that exhibited two interactions with Leu 141 and Asp 207 by two hydrogen bond of 2.96 and 2.22 Å, respectively (Fig. 7) which may explain its promising cytotoxic activity.

## Conclusion

In conclusion, synthesis, structural elucidation, antimicrobial and anticancer activities of a new series of *N*-(2,6-dimethoxypyrimidin-4-yl)-4-(3-(aryl) thioureido)benzenesulfonamides **3a**–**e** and **4a**–**d** were reported. Compound **4a** was the most active compound against Gram positive bacteria (*B. subtilis* and *S. pneumoniae*), Gram negative bacteria (*P. aeruginosa* and *E. coli*) and fungi (*A. fumigatus*). Interestingly, compounds **4c** and **4d** were selective to Gram positive bacteria. Compound 4a was the most active compound in cytotoxic assay against breast cancer cell line (MCF-7) and hepatic cancer cell line (HepG2). Compound **4a** was more active than the standard drug 5-flourouracil in case of hepatic cancer cell line (HepG2). Molecular docking of compound 4a on the active site of mitogen activated kinase (MK-2) revealed good amino acid interactions.

## Experimental

### General chemistry

Melting points (uncorrected) were determined in an open capillary in a Gallenkamp melting point apparatus (Sanyo Gallenkamp, UK). Pre coated silica gel plates (Kieselgel 0.25 mm, 60 F254, Merck, Germany) were used for thin layer chromatography. A developing solvent system of chloroform/methanol (8:2) was used and the spots were detected by ultraviolet light. IR spectra (KBr disc) were recorded using an FT-IR spectrophotometer (Perkin Elmer, USA). ^1^H NMR spectra were scanned on an NMR spectrophotometer (Bruker AXS Inc., Switzerland), operating at 500 MHz for ^1^H- and 125.76 MHz for ^13^C NMR. Chemical shifts are expressed in δ values (ppm) relative to TMS as an internal standard, using DMSO-*d*_*6*_ as a solvent. Elemental analyses were done on a model 2400 CHNSO analyser (Perkin Elmer, USA). All values were within ±0.4% of the theoretical values. All reagents used were of AR grade.

### General procedure for *N*-(2,6-dimethoxypyrimidin-4-yl)-4-(3- (aryl) thioureido)benzenesulfonamides 3a–e and 4a–d

To a mixture of isothiocyanatobenzenesulfonamide **2** (0.01 mol) and fluorinated aromatic amine (0.01 mol) in dioxane (30 mL), triethylamine (0.1 mL) was added. The reaction mixture was heated under reflux for 1 h. The solvent was removed by evaporation under reduced pressure and the remainder was left to cool. The solid product so formed was collected by filtration, washed with petroleum ether (bp 40–60 °C) and the crude product recrystallized from ethanol to afford thiourea derivatives.

#### *N*-(2, 6-Dimethoxy-pyrimidin-4-yl)-4-[3-(2-fluoro-phenyl)-thioureido]enzene-sulfonamide (**3a**)

This compound was obtained as brown crystals from ethanol; yield 83%; m.p. 348.1 °C. IR: 3444, 3354, 3232 (NH), 3091 (arom.), 2956, 2810 (aliph.), 1568 (CN), 1361, 1180 (SO_2_), 1265 (CS). ^1^HNMR: δ 3.81, 3.84 [2 s, 6H, 2OCH_3_], 5.9 [s, 1H, H- pyrimidine], 6.7–8.0 [m, 8H, Ar–H], 10.2 [s, 1H, SO_2_NH], 11.8, 14.0 [2 s, 2H, 2NH]; ^13^C-NMR: 55.2, 55.8, 87.0, 113.1, 122.9 (3), 128.4 (2), 129.1 (2), 131.5, 133.8, 140.0, 162.7 (2), 165.3, 170.2, 182.0. Anal. Calcd. for C_19_H_18_FN_5_O_4_S_2_:C, 49.24%; H, 3.91%; F, 4.10%; N, 15.11%; S, 13.84%. Found: C, 49.30%; H, 3.80%; F, 4.20%; N, 15.12%; S, 13.84%.

#### *N*-(2,6-Dimethoxy-pyrimidin-4-yl)-4-[3-(3-fluoro-phenyl)-thioureido]benzene-sulfonamide (**3b**)

This compound was obtained as brown crystals from ethanol; yield 87%; m.p. 133.9 °C. IR: 3446, 3365 (NH), 3100 (arom.), 2978, 2831 (aliph.), 1635 (CN), 1396, 1182 (SO_2_), 1274 (CS). ^1^HNMR: δ 3.80, 3.83 [2 s, 6H, 2OCH_3_], 6.4 [s, 1H, H -pyrimidine], 6.9–8.4 [m, 8H, Ar–H], 9.8 [s, 1H, SO_2_NH], 11.7 [s, 2H, 2NH]; ^13^C-NMR: 55.4, 56.5, 80.0, 112.7, 120.6, 121.2, 123.7 (2), 128.1 (2), 132.6 (2), 137.0, 142.6, 163.0, 164.5, 164.9, 178.4, 179.8. Anal. Calcd. for C_19_H_18_FN_5_O_4_S_2_:C, 49.24%; H, 3.91%; F, 4.10%; N, 15.11%; S, 13.84%. Found: C, 49.50%; H, 3.60%; F, 4.50%; N, 15.40%; S, 13.80%.

#### *N*-(2,6-Dimethoxy-pyrimidin-4-yl)-4-[3-(4-fluoro-phenyl)-thioureido]benzenesul-fonamide (**3c**)

This compound was obtained as brown crystals from ethanol; yield 90%; m.p. > 360 °C. IR: 3442, 3186 (NH), 3062 (arom.), 2941, 2839 (aliph.), 1624 (CN), 1382, 1132 (SO_2_), 1271 (CS). ^1^H-NMR: δ 3.72, 3.75 [2 s, 6H, 2OCH_3_], 6.4 [s, 1H, H- pyrimidine], 6.9–8.8 [m, 8H, Ar–H], 9.7 [s, 1H, SO_2_NH], 11.4 [s, 2H, 2NH]. ^13^C-NMR: δ 55.4, 55.7, 84.1, 113.6 (2), 121.0 (2), 127.5 (2), 129.6 (2), 132.7, 133.8, 140.0, 158.6, 161.9, 165.8, 170.9, 182.3. Anal. Calcd. For C_19_H_18_FN_5_O_4_S_2_:C, 49.24%; H, 3.91%; F, 4.10%; N, 15.11%; S, 13.84%. Found: C, 49.10%; H, 3.80%; N, 15.30%; S, 13.70%.

#### *N*-(2,6-Dimethoxy-pyrimidin-4-yl)-4-[3-(3-fluoro-4-methoxy-phenyl)-thioureido]benzenesulfonamide (**3d**)

This compound was obtained as yellow crystals from ethanol; yield 88%; m.p. 289 °C. IR: 3421, 3230 (NH), 3086 (arom.), 2910, 2841 (aliph.), 1624 (CN), 1400, 1128 (SO_2_), 1286 (CS). ^1^H-NMR: δ 3.84, 3.91, 3.96 [3 s, 9H, 3OCH_3_], 6.4 [s, 1H, H-pyrimidine], 6.9–8.4 [m, 7H, Ar–H], 8.9 [s, 1H, SO_2_NH], 11.5 [s, 2H, 2NH];^13^C-NMR:δ 55.4, 55.6, 57.4, 79.6, 117.3 (2), 121.7 (2), 122.0, 129.7(2), 130.1, 134.7, 139.8, 140.9, 150.8, 160.2, 162.9, 172.0, −183.3. Anal. Calcd. For C_20_H_20_FN_5_O_5_S_2_:C, 48.67%; H, 4.08%; F, 3.85%; N, 14.19%; O, 16.21%; S, 12.99%. Found: C, 48.50%; H, 4.10%; F, 3.30%; N, 14.20%; S, 12.70%.

#### *N*-(2,6-Dimethoxy-pyrimidin-4-yl)-4-[3-(4-fluoro-2-nitro-phenyl)-thioureido]benzenesulfonamide (**3e**)

This compound was obtained as yellow crystals from ethanol; yield 86%; m.p. 143.5 °C. IR: 3421, 3197 (NH), 3062 (arom.), 2936, 2839 (aliph.), 1625 (CN), 1384, 1132 (SO_2_), 1269 (CS). ^1^H-NMR: δ3.80, 3.84 [2 s, 6H, 2OCH_3_], 6.5 [s, 1H, H-pyrimidine], 6.9–8.4 [m, 7H, Ar–H], 9.8 [s, 1H, SO_2_NH], 10.4, 11.0 [2 s, 2H, 2NH]. ^13^C-NMR: 55.4, 56.5, 79.8, 112.7, 121.4, 125.5 (2), 127.8 (2), 128.1 (2), 133.6, 144.0, 144.9, 153.1, 155.3, 165.7, 170.2, 178.8. Anal. Calcd. For C_19_H_19_FN_6_O_6_S_2_: C, 44.70%; H, 3.75%; F, 3.72%; N, 16.46%; S, 12.56%. Found: C, 44.60%; H, 3.50%; F, 3.70%; N, 16.40%; S, 12. 60%.

#### *N*-(2,6-Dimethoxy-pyrimidin-4-yl)-4-[3-(2,3,5,6-tetrafluoro-pyridin-4-yl)-thioureido]benzenesulfonamide (**4a**)

This compound was obtained as brown crystals from ethanol; yield 87%; m.p. 281.7 °C.IR: 3400, 3192 (NH), 3057 (arom.), 2931, 2871 (aliph.), 1620 (CN), 1386, 1128 (SO_2_), 1257 (CS). ^1^H-NMR:δ 3.80, 3.84 [2 s, 6H, 2OCH_3_], 6.1 [s, 1H, H-pyrimidine], 6.7–8.2 [m, 4H, Ar–H],10.3 [s, 1H, SO_2_NH], 11.7 [s, 2H, 2NH]; ^13^C-NMR: δ 55.4, 55.6, 81.2, 121.8 (2),128.7 (2), 130.2(2), 137.1, 140.8, 143.0, 144.5 (2), 161.4, 161.9, 169.7, 184.6. Anal. Calcd. For C_18_H_14_F_4_N_6_O_4_S_2_:C, 41.60%; H, 2.42%; F, 14.60%; N, 16.30%; S, 12.40%. Found: C, 41.60%; H, 2.42%; F, 14.60%; N, 16.30%; S, 12.40%.

#### *N*-(2,6-Dimethoxy-pyrimidin-4-yl)-4-[3-(5-trifluoromethyl-[1, 3, 4]thiadiazol-2-yl)-thioureido]-benzenesulfonamide (**4b**)

This compound was obtained as brown crystals from ethanol; yield 86%; m.p. 276.7 °C. IR: 3400, 3201(NH), 3045 (arom.), 2923, 2801 (aliph.), 1622 (CN),1386,1130 (SO_2_),1323 (CS). ^1^H-NMR: δ 3.80, 3.81 [2 s, 6H, 2OCH_3_], 6.1 [s, 1H, H-pyrimidine],6.9–8.4 [m, 4H, Ar–H], 10.1 [s, 1H, SO_2_NH], 10.3, 12.0 [2 s,2H, 2NH]; ^13^C-NMR: 56.2, 56.7, 81.6, 117.2, 120.8 (2), 130.7 (2), 134.6, 143.0, 151.1, 154.6, 161.7, 163.9, 173.0, 182.8. Anal. Calcd. for C_16_H_14_F_3_N_7_O_4_S_3_:C, 36.85%; H, 2.71%; F, 10.93%; N, 18.80%; S, 18.45%. Found: C, 36.70%; H, 2.90%; F, 10.70%; N, 18.90%; S, 18.90%.

#### *N*-(2,6-Dimethoxy-pyrimidin-4-yl)-4-[3-(6-fluoro-benzothiazol-2-yl)-thioureido]-benzenesulfonamide (**4c**)

This compound was obtained as brown crystals from ethanol; yield 92%; m.p.179.7 °C. IR: 3415, 3310 (NH), 3075 (arom.), 2978, 2841 (aliph.), 618 (CN), 1311, 1195 (SO_2_),1274 (CS).^1^H-NMR: δ 3.64, 3.66 [2 s, 6H, 2OCH_3_], 6.5 [s, 1H, H-pyrimidine], 6.9–8.5 [m, 7H, Ar–H],9.7[s,1H, SO_2_NH],11.8, 12.4 [2 s, 2H, 2NH].^13^C-NMR: 55.4, 55.8, 83.7, 109.1, 112.0, 119.2, 123.7 (2), 128.1 (2), 132.8, 134.2, 139.9, 147.1, 156.6, 157.1, 165.3, 174.0, 175.1, 183.2. Anal. Calcd. for C_20_H_17_FN_6_O_4_S_3_: C, 46.14%; H, 3.29%; F, 3.65%; N, 16.14%; S, 18.48%. Found: C, 46.20%; H, 3.40%; F, 3.60%; N, 16.30%; S, 18.48%.

#### *N*-(2,6-Dimethoxy-pyrimidin-4-yl)-4-[3-(4-trifluoromethyl-2-oxo-2H-chromen-7-yl)-thioureido]-benzenesulfonamide (**4d**)

This compound was obtained as brown crystals from ethanol; yield 81%; m.p.209.7 °C. IR: 3454, 3361, 3250 (NH), 3100 (arom.), 2970, 2861 (aliph.), 1708 (CO), 1635 (CN),1350,1165 (SO_2_), 1286 (CS). ^1^H-NMR: 3.80, 3.82 [2 s, 6H, 2OCH_3_], 6.3 [s, 1H, H -pyrimidine], 6.8–8.0 [m, 8H, Ar–H + H -chromene], 9.8 [s, 1H, SO_2_NH], 11.4 [s,2H, 2NH]; ^13^C-NMR: 55.4, 55.6, 80.6, 107.2, 112.6 (2), 121.3 (2), 126.1 (3), 129.0 (2), 133.0 (2), 140.5, 154.5 (2), 157.0, 159.8, 165.2, 174.6, 180.3. Anal. Calcd. for C_23_H_18_F_3_N_5_O_6_S_2_: C, 47.50%; H, 3.12%; F, 9.80%; N, 12.04%; S, 11.03%. Found:C, 47.70%; H, 3.50%; F, 9.70%; N, 12.30%; S, 11.20%.

### Antimicrobial activity assay

All microbial strains were provided from culture collection of the Regional Center for Mycology and Biotechnology (RCMB), Al-Azhar University, Cairo, Egypt. The antimicrobial activity was investigated on a dozen of newly synthesized compounds in order to increase the selectivity of these derivatives towards test microorganisms using well diffusion method [42]. Briefly, 100 μL of the test bacteria/fungi were grown in 10 mL of fresh media until they reached a count of approximately 10^8^ cells/mL for bacteria or 10^5^ cells/mL for fungi. One hundred µL of each sample (at 1 mg/mL) was added to each well (10 mm diameter holes cut in the agar gel). The plates were incubated for 24–48 h at 37 °C (for bacteria and yeast) and for 48 h at 28 °C (for filamentous fungi). After incubation, the microorganism’s growth was observed. Ampicillin and gentamycin were used as standard antibacterial drugs while amphotricin B was used as standard antifungal drug. The resulting inhibition zone diameters were measured in millimeters and used as criterion for the antimicrobial activity. If an organism is placed on the agar it will not grow in the area around the well if it is susceptible to the chemical. This area of no growth around the disc is known as a zone of inhibition. The size of the clear zone is proportional to the inhibitory action of the compound under investigation. Solvent controls (DMSO) were included in every experiment as negative controls. DMSO was used for dissolving the tested compounds and showed no inhibition zones, confirming that it has no influence on growth of the tested microorganisms. The active compounds were further investigated to determine their antimicrobial activity expressed in terms of minimum inhibitory concentration (MIC) using the modified agar well diffusion method that mentioned above. Concentrations between 0.1 and 1000 µg/mL of each active compound were tested and compared with standard drugs. The MIC was then determined as the lowest concentration inhibiting growth of the organism after 24–48 h.

### Antitumor activity assay

The tested human carcinoma cell lines were obtained from the American Type Culture Collection (ATCC, Rockville, MD). The cells were grown on RPMI-1640 medium supplemented with 10% inactivated fetal calf serum and 50 µg/mL gentamycin. The cells were maintained at 37 °C in a humidified atmosphere with 5% CO_2_ and were sub-cultured two to three times a week.

For antitumor assays, the tumor cell lines were suspended in medium at cell density of 5 × 10^4^ cells/well in Corning^®^ 96-well tissue culture plates, then incubated for 24 h. The tested compounds were then added into 96-well plates (six replicates) to achieve eight concentrations for each compound. Six vehicle controls with media or 0.5% DMSO were run for each 96 well plate as a control. After incubating for 24 h, the numbers of viable cells were determined by the MTT assay [43, 44]. Briefly, the media was removed from the 96-well plate and replaced with 100 µL of fresh culture RPMI 1640 medium without phenol red then 10 µL of the 12 mM MTT stock solution (5 mg of MTT in 1 mL of PBS) to each well including the untreated controls. The 96 well plates were then incubated at 37 °C and 5% CO_2_ for 4 h. An 85 µL aliquot of the media was removed from the wells, and 50 µL of DMSO was added to each well and mixed thoroughly with the pipette and incubated at 37 °C for 10 min. Then, the optical density was measured at 590 nm with the microplate reader (SunRise, TECAN, Inc, USA) to determine the number of viable cells and the percentage of viability was calculated as [1−(ODt/ODc)] × 100% where ODt is the mean optical density of wells treated with the tested sample and ODc is the mean optical density of untreated cells. The relation between surviving cells and drug concentration is plotted to get the survival curve of each tumor cell line after treatment with the specified compound. The 50% inhibitory concentration (IC_50_), the concentration required to cause toxic effects in 50% of intact cells, was estimated from graphic plots of the dose response curve for each conc. using Graphpad Prism software (San Diego, CA, USA) [31].

### Molecular docking

All the molecular modeling studies were carried out on an Intel Pentium 1.6 GHz processor, 512 MB memory with Windows XP operating system using Molecular Operating Environment (MOE, 10.2008) software. All the minimizations were performed with MOE until a RMSD gradient of 0.05 kcal mol^−1^ Å^−1^ with MMFF94X force field and the partial charges were automatically calculated. The protein data bank file (PDB:3WI6) was selected for this purpose. The file contains MK-2 enzyme Co-crystalized with a ligand obtained from protein data bank. The enzyme was prepared for docking studies where: (i) Ligand molecule was removed from the enzyme active site. (ii) Hydrogen atoms were added to the structure with their standard geometry. (iii) MOE Alpha Site Finder was used for the active sites search in the enzyme structure and dummy atoms were created from the obtained alpha spheres. (iv) The obtained model was then used in predicting the ligand enzymes interactions at the active site.
